# Hudson River Association of Dental Surgeons

**Published:** 1869-08

**Authors:** 


### HUDSON RIVER ASSOCIATION OF DENTAL SURGEONS.

The regular annual meeting of this Association took place Wednesday, May 12th, in Odd Fellows' Hall, Liberty street, Poughkeepsie. It is composed of Dentists residing in the towns on and adjacent to the Hudson River. It is one of the largest and most important Dental Societies in this State, numbering nearly one hundred members. At the morning session but little business was done except to listen to the retiring President's (Dr. Fuller) annual address, in which he reviewed the progress the profession had made from its infancy to its present standing.

The afternoon session was devoted to the election of officers for the ensuing year and discussing the regular subject selected at the last meeting, viz.: "Treatment of Exposed Nerves."

Dr. Houghton and Dr. Straw read very able essays on the subject, and received the thanks of the Association. The subject was thoroughly discussed, and afforded much information to the Dentists present.

In the evening the subject of Mechanical Dentistry came up for discussion and was participated in by nearly all the members present. The rubber work and Folsom's Patent was pretty well discussed and their weak points thoroughly ventilated.

Dr. Houghton suggested that the Association publish a Dental Journal for the benefit of the public. The matter was referred to a committee of three, consisting of Drs. Fuller, Straw and Royce, to report at the next meeting.

The following are the names of the officers elected:

President, Dr. L. S. Straw, Newburg; 1st Vice President, Dr. T. C. Royce, Middletown; 2d Vice President, Dr. W. B. Van Vleeck, Hudson; Recording Secretary, Dr. T. W. DuBois, Poughkeepsie; Corresponding Secretary, Dr. Chas. L. Houghton, Poughkeepsie; Treasurer, Dr. Chas. F. Allan, Newburgh; Executive Committee, Dr. J. H. Mann, Poughkeepsie, Dr. H. Smith, Hudson, Dr. H. M. Fressell, Kingston, Dr. W. E. Birdsall, Kingston, Dr. J. Shelden, Hudson.

Drs. Houghton, Birdsall, Allen, Du Bois and Van Duser were elected delegates to represent the Society at the American Dental Association, at Saratoga, in August.

